# Tolerance Induction in Liver

**Published:** 2015-05-01

**Authors:** M. H. Karimi, B. Geramizadeh, S. A. Malek-Hosseini

**Affiliations:** Transplant Research Center, Shiraz University of Medical Science, Shiraz, Iran

**Keywords:** Liver transplantation, Tolerance, Regulatory T cells

## Abstract

Liver is an exclusive anatomical and immunological organ that displays a considerable tolerance effect. Liver allograft acceptance is shown to occur spontaneously within different species. Although in human transplant patients tolerance is rarely seen, the severity level and cellular mechanisms of transplant rejection vary. Non-paranchymal liver cells, including Kupffer cells, liver sinusoidal endothelial cells, hepatic stellate cells, and resident dendritic cells may participate in liver tolerogenicity. The mentioned cells secret anti-inflammatory cytokines such as TGF-β and IL-10 and express negative co-stimulatory molecules like PD-L1 to mediate immunosuppression. Other mechanisms such as microchimerism, soluble major histocompatibility complex and regulatory T cells may take part in tolerance induction. Understanding the mechanisms involved in liver transplant rejection/tolerance helps us to improve therapeutic options to induce hepatic tolerance.

## INTRODUCTION

The liver has gradually been accepted as an immune organ [1]. As a unique immunological and metabolic organ, liver confronts several antigens in blood via the gastrointestinal tract. While passing through sinusoids, antigens are cleaned by lymphocytes. Various types of antigen presenting cells (APCs) exist in the liver to capture the antigens circulating in blood. Due to the slow blood flow in sinosoids, APCs have ample opportunity to process and present the antigens to circulating lymphocytes for their clearance. The ability of the liver to induce immune responses to pathogens and antigen-specific tolerance arise from direct or indirect activation of lymphocytes [2].

In many situations, the outcome of T cell stimulation by the liver cells is local with systemic immune tolerance. Studies have demonstrated that liver allograft is not only being well accepted, but also can assist the acceptance of skin grafts from the liver donor, while a third party skin graft is rapidly rejected [3]. The liver tolerance effect was first described in 1969 by demonstrating that without immunosuppression liver allograft was accepted across major histocompatibility complex (MHC) mismatch in pig [1]. Subsequent studies confirmed this phenomenon in other species and indicated that recipients of liver allograft could even accept donor-specific non-hepatic allograft, such as heart and skin, whereas third party grafts were rejected [3]. Moreover, ongoing rejection of previously transplanted organs could be reversed by liver allograft. Combined transplantation of human liver together with kidney or lung of the same donor maintains the liver graft survival and prevents its rejection [3]. Therefore, it is assumed that the liver contains specialized cells which mediate the tolerogenic properties of the organ. Understanding these mechanisms can help us to develop specific immune therapies for both augmentation and for tolerance failure. 

## LIVER ANTIGEN PRESENTING CELLS AND LYMPHOCYTES

Liver Dendritic Cells

Dendritic cells (DCs), as the main professional APCs, migrate to draining lymph nodes near the site of infection to exploit immune activation and T lymphocytes responses, while resident liver DCs located around the portal tracts and central veins do not enter draining lymph nodes [4]. Containing as much as plasmacytoid as myeloid DC, the resident liver DCs display an immature phenotype that results in liver tolerogenic properties [1]. MHC class I (MHC-I) and co-stimulatory molecules such as CD40, CD80 and CD86 are expressed in low levels in the liver [2]. Liver DC may inhibit cytokine production and proliferation of effector T cells via programmed death ligand 1 (PD-L1) and cytotoxic T lymphocyte antigen 4 (CTLA-4) [4]. Liver originated TGF-β and IL-10 as well as CD4+, CD25+, FOXP3+ regulatory T cells take part in the tolerogenic phenotype of hepatic DC [1]. CCR5 up-regulation and CCR7 down-regulation by DC are increased by IL-10 generated in the liver. Another resident DC population has been shown in the kidney with the characteristic of producing IL-10 for induction of CD4+ T cells. Although, renal DC displays the immature phenotype, it does not make the kidney become an immunoprivileged organ as the liver [1]. IL-12 is generated by resident liver DC, but it sounds as if liver DCs are more tolerogenic than other tissues APCs [3]. 

Kupffer Cells (KC)

KCs are tissue resident macrophages, supposed to form 20% of non-parenchymal cells within the sinusoidal lumen in the periportal liver area. Deriving from bone marrow precursors and blood monocytes, KCs can clear endotoxin, phagocyte microbes and apoptotic cells through the space of Disse and also through the release of pro-inflammatory cytokines such as IL-1, IL-6, IL-12, IL-18, TNF-α and IFN-γ [1]. IL-1, IL-6 and TNF-α induce neutrophilic granulocytes infiltration to remove bacteria. TNF-α induces apoptosis of hepatocytes in pathological condition. Simultaneously, TNF-α secretion in low concentrations induced by physiological levels of endotoxins leads to hepatocytes resistance to apoptosis [5]. IL-12 and IL-18 activate NK cells to produce IFN-γ with anti-viral effects, while IL-10 can reduce IL-6 and TNF-α production [6]. MHC class II expression and low levels of co-stimulatory molecules (CD80 and CD86) indicate that KCs are APCs that can activate CD4+ T cells. At the same time, CD4+ T cell activity may be hampered by prostaglandins and nitric oxide produced by KCs [7]. In response to LPS, KC produced pro-inflammatory cytokines and significant amounts of IL-10 [8]. One of the symptoms of end-stage liver failure is loss of KCs clearance function and persistence of infections. In liver transplantations, KCs behave as APCs, which identify and interact with recipient T cells that migrate to the transplanted organ. KCs can commence T cell apoptosis via Fas/FasL pathway, which can be arrested by anti-FasL antibodies. Increasing NF-κβ activity in KCs leads to higher levels of FasL and IL-4 in KC to promote apoptosis of T cells. Using gadolinium trichloride destroys KCs and helps to block T cell apoptosis [9].

Liver Sinusoidal Endothelial Cells (LSECs) 

LSECs comprise 50% of non-parenchymal liver cells. Unlike complete barrier of vascular endothelial cells, which isolate hepatic tissue from the blood, sinusoidal fenestrated endothelium establishes a porous layer which enables hepatocytes and T lymphocytes to contact. LSECs express two kinds of molecules to ease antigen processing and presentation (like APCs). The molecules induce antigen uptake including mannose receptor, scavenger receptor, and second antigen-presenting molecules like MHC-I, MHC-II, CD40, CD80, and CD86. LSECs activate effector/memory T cells, mainly CD4+ T cells, proliferated by LSECs stimulation. IL-10 and PGE2 produced by KCs can down-modulate the activity of effector/memory T cells, which is terminated by T cell tolerance and hepatic tolerance [1]. A decrease in MHC-II, CD80, CD86 expression and mannose receptor activity by IL-10 secretion has been reported [1]. PD-L1 is shown to be responsible for tolerance induction in LSECs [10]. Venous blood constantly provides physiological levels of endotoxin, which can promote IL-10 secretion from LSEC and KCs and as well as down-modulation of CD4+ T cell activation by LSEC via down-regulation of MHC-II, CD80, and CD86 expression [11]. Endotoxin activates APC through TLR4 to enhance T cell activation. Activation of TLR3 and TLR9 destroys the immune tolerance of the liver and induces CD8+ T cell-mediated hepatitis. LSEC are able to cross-present exogenous antigen towards CD8+ T cells and leads to CD8+ T cell tolerance versus immunity [12]. Tolerance induction is correlated with PD-L1 induction by LSEC [10].

Hepatic Stellate Cells

Hepatic stellate cells or Ito cells (also known as fat-storing cells), with an important role in the ability to store vitamin A and hepatic fibrosis, are distinguished as professional liver APCs. Ito cells present lipid antigens to natural killer cells, while protein and lipid antigens are presented to CD1, MHC-I, MHC-II, restricted T cells, and CD8+ and CD4+ T cells. Unlike other hepatic cells inducing hepatic tolerance during microbial infection, stellate cells elicit specific responses towards lipid and protein antigens as potent liver APCs [13]. Ito cells display efficient immunomodulatory potential by B7-H1 mediated apoptosis in T cells and express PD-L1 [14]. If T cells induced by Ito cells secrete TGF-β, they mainly differentiate to FOXP3+ Tregs. CD4+ T cells can be converted to induced regulatory T cells (iTregs) by vitamin A-derived retinoic acid and/or TGF-β. Although TGF-β is the most potent mediator of fibrogenesis in the liver, it shows anti-inflammatory cytokine's features [15]. Ito cells produce IL-1, IL-6, and IL-15, but tolerogenic properties have been shown in islet allograft protection by stellate cells transplantation [16, 17].

Hepatocytes

Hepatocytes are assumed to be about 70% of all hepatic cells. This parenchymal cell population is outstanding for its both immunomodulatory functions and metabolic roles. Hepatocytes immunoregulatory functions may be due to their function as APCs. They express high levels of MHC-I to present antigen to both CD8+ T lymphocytes and NKT cells [1, 18]. Hepatocytes and T lymphocytes interact with each other but lymphocyte extensions pass through fenestrations in the space of Disse in inflammatory conditions such as clinical hepatitis, viral or autoimmune diseases and make parenchymal cells express MHC-II [1, 18]. By expressing MHC-II, hepatocytes show their ability to activate CD4+ T cells as APCs [19]. PD-L1 and PD-L2 are negative co-stimulatory molecules expressed by potent APCs. PD-L2 is just expressed by APCs while PD-L1 on the hepatocytes facilitates hepatocyte damages in experimental autoimmune hepatitis (EAH). Type I and II interferons and viral infections induce PDL1 expression on hepatocytes [20]. 

Because PD-L1 is also inducible by IL-10 and IL-10 is a dominant cytokine in the liver produced by resident DCs, KCs, and LSECs, it seems that PD-L1 induction in hepatocytes in response to inflammation contributes to the tolerogenic effect mediated by these cells [20]. These data suggest that hepatocytes in cooperation with activated LSECs, KCs, and HSCs mediate T cell tolerance in the liver. Since each of the liver resident cells might induce—after initial activation—a state of non-responsiveness in either CD4+ or CD8+ T cells, all of them might facilitate transformation of CD4+ T cells to iTregs probably in the presence of naturally occurring regulatory T cells (nTregs) *in vivo*.

Regulatory T Cells

Recently, different lymphocyte subsets have been shown to have regulatory functions: Th3 cells, Tr1 cells and CD4+ CD25+ FOXP3+ T cells (Treg). Naturally occurring regulatory T cells suppress activation and effector functions of various cell types including CD4+ and CD8+ T cells, NK cells, NK T cells, DCs, and B cells. Regulatory T cells are pivotal for retention of peripheral tolerance and lowering immune responses. By cell contact, Treg induces suppression in T cell activation *in vitro*. The regulatory function of Treg might be done by *in vivo* IL-10 and TGF-β secretion. Treg co-inhibitory molecules including PDL-1, CTLA-4 and glucocorticoid-induced TNF receptor family-related gene (GITR) have a critical role in preventing T cell-mediated inflammatory diseases [20, 21]. A CD4+ CD25+ FOXP3+ Treg is a regulatory T cell, with a pivotal role in autoimmunity, cancer, and transplantation. It has been shown that generation of Treg from non-Treg *in vitro* is TGF-β and IL-10 dependent [22]. 

It seems that Treg is generated in periphery and the thymus, while liver has developed several mechanisms for induction of hepatic tolerance and its maintenance. Comparing splenic ones, hepatic Treg expresses higher levels of FOXP3+, CTLA-4, GITR, and CD103. The association of autoimmune hepatitis and occurrence of primary biliary cirrhosis with decrease in Treg, has been proposed repeatedly [23, 24]. 

T cell mitogenic lectin concavalin A (ConA)-induced inflammatory injury in the liver leads to induction of hepatic tolerance by higher secretion of IL-10, down-modulation of TNF-α, IL-6, IL-12 , IFN-γ, and IL-17 as well as serum transaminase activity reduction. So, CD4+ CD25+ FOXP3+ T cells and KCs tolerance induction in the liver is associated with IL-10 release [25].

CD25+ depletion leads to liver injury induced by ConA, while CD25+ transfer improves the severity of this damage. Also, ConA liver inflammation is increased by TGF-β signaling pathway blockade, so it is proposed that anti-inflammatory activity of Treg is dependent on TGF-β [26].

Expression of an autoantigen into the liver promotes antigen specific Treg production to protect people from autoimmune diseases and induces active tolerance. Conversion of conventional CD4+ T cells to autoantigen-specific CD4+ CD25+ FOXP3+ Treg needs autoantigen presentation to the liver and TGF-β secretion. This can be a clue to finding the treatment strategy for hepatic injuries [3]. 

In chronic viral hepatitis, different regulatory T cells, such as Treg, IL-10 or TGF-β-secreting CD4+ and CD8+ T cells can help to impair anti-viral immune responses. Treg presents 30%–50% of hepatic CD4+ T cells in chronically HCV-infected patients. Therefore, persistence of HCV infection is seen because of Treg attenuating HCV-specific T cell responses [27].

## MECHANISMS OF HEPATIC CD4+ T CELL TOLERANCE

Hepatic immune deviation

A Th1 cell is a pro-inflammatory T cell secreting IFN-γ. Various T helper cell lineages are differentiated from the naive T cell. IL-12 can promote the differentiation into Th1 helper. Th1 secreting IFN-γ is in favor of cellular immunity while Th2 cells are characterized by IL-4 production and have a major role in humoral immunity. IL-4 induces Th2 differentiation. Tregs are another type of T cells suppressing effector T cells. TGF-β is responsible for differentiation into CD4+ CD25+ FOXP3+ Tregs. Th17, a newly discovered T helper lineage induced by IL-6 and IL-21, is important in autoimmunity and inflammatory diseases [28]. Th1 and Th17 responses play a major role in many inflammatory liver conditions. Liver Th17 infiltration in murine model of biliary and human cirrhosis patients has been significantly increased. Also, CD4+ T cells activated by the liver non-parenchymal cells secrete more IL-17 than those activated in the presence of the spleen T cells [29]. Th17 is related to alcoholic liver disease and autoimmune hepatitis. Also, IL-17 is produced by intrahepatic TCRγδ T cells, which show the protective role against the Listeria infection of the liver [18]. Th1 responses seem to play a role in acute liver injury and autoimmune liver disease [30, 31]. Antigen presentation by liver APCs may cause immune deviation and differentiation of non-Th1 or Th17 CD4 cells. A normal liver can inhibit an extensive immune response and keep the liver tolerance. Naive CD4+ T cells differentiating to Th2 phenotype, synthesize mainly IL-4 and IL-10. IFN-γ-producing cells (Th1) are selectively suppressed by LSEC to promote IL-4-secreting Th2 cells [32]. *In vivo*, Th1 cells lose their ability to produce cytokines, while IL-4 expression is maintained within Th2 cells [32]. Liver DC, as a potent APC, induces immune deviation by promoting Th1 cells apoptosis and Th2 responses. Because of hepatic cytokine milieu liver DC maturation is different from that of other organs. Culturing human monocyte liver cells leads to IL-10-producing DC, which develops Th2 cells responses to present their anti-inflammatory effects [33]. Also, plasmacytoid DC leads to Treg production. Human liver DCs are more tolerogenic than immunogenic; they promote Th2 generation and Treg development in an IL-10-dependent manner [16]. MHC-II expressing hepatocyte can also induce immune deviation. Naive CD4+ T cells activated by hepatocytes lead to Th2 differentiation, but not Th1 cells IFN-γ production [34]. *In vivo*, hepatocytes MHC-II was shown to be related to impaired viral Th1 responses and clearance of lymphocytic choriomeningitis virus (LCMV). Immune deviation may undergo chronic HCV infection. In acute HCV infection, Th1 responses are developed and IL-2, IFN-γ productions are promoted so that the patients with Th2-dominated responses are more prone to chronic diseases. In stable infections, impaired CD4+ T cell function may secrete IFN-γ but no IL-2 production [35, 36]. Immune deviation of CD4+ T cell may play a key role in hepatic tolerance. Immune deviation can also defect T cell responses to LCMV and HCV virus. Thus, immune deviation facilitates hepatic tolerance and is involved in defective T cell responses to hepatotropic viruses.

Inhibitory T Cell Stimulation: CTLA-4 and PD-1/PD-L1

PD-1 and CTLA-4 sustain peripheral T cell tolerance by various mechanisms. CTLA-4 shares the ligands CD80 and CD86 together with CD28 and is rapidly expressed on the cell surface upon T cell activation. CTLA-4 seems to be critical for short-term tolerance in the peripheral organs [37]. Programmed death-1 receptor is an inhibitory molecule expressed on activated T cells; its ligand, PDL-1, is expressed on leukocytes, non-hematopoietic cells, and non-lymphoid tissues, while PD-L2 is expressed on DC and monocytes. CTLA-4 is believed to limit T cell responses early after stimulation in lymphatic tissues. PD-1 is shown to prevent long-term T cell responses after stimulation [37]. In the liver, LSEC, KC, stellate cells and hepatocytes express PD-L1. Since PD-1/PD-L1 co-inhibitory ligation modulates the immune responses, in the absence of PD-1 signaling PD-1 (–/–) mice effector T cells proliferation is induced, so in contrast to the wild mice, PD-1 (–/–) animals shows more severe hepatitis in the early stage of infection, but rapid clearance of the virus. 

Functional exhaustion, due to impaired T cell responses, leads to chronic viral persistence. Failure of T cell response leads to impaired cytokine production and proliferation even after long stimulation of antigens [38].

One of the major causes of chronic viral infections is PD-1/PD-L1 interactions, which are associated to exhaustion of CD8+ T cells in LCMV, HCV, HBC and HIV viral infections. Therefore, blocking PD-1/ PD-L1 may be a therapeutic modality for treating viral infections [39]. Activated CD8+ T cells depletion in the liver is associated with hepatic PD-L1. That is why PD-L1 (–/–) mice liver cells are accumulated by CD8+ T cells and promote ConA inflammatory induction in hepatic disorders [40]. The role of PD-1 in liver immunity is established for suppression of CD8+ T cells’ activity during infection persistence. CD4+ T cell unresponsiveness is associated with other mechanisms including the promotion of CD4+ and CD8+ T cell apoptosis based on PD-L1 by activated hepatic stellate cells [41]. In contrast, CTLA-4 mechanism in the immune response is less investigated. CTLA-4 does not change in the peripheral blood of patients suffering from persistent HCV infection, while it is up-regulated on PD-1-positive CD8+ T cells of the liver. Therefore, efficient recovery of intrahepatic T cell function requires both, PD-1 and CTLA-4 blockade and is independent of CD4 T cells including Treg [42]. 

Inhibitory Cytokines: IL-10 and TGF-β

Various cytokines including IL-10 and TGF-β produced by different cell types of the liver are believed to regulate oral tolerance. IL-10 has a key role in oral tolerance induction in transgenic mice with maintained hepatocytes specific expression of rat IL-10. Additionally, secretion of IL-10 in the liver of transgenic mice appears to be more effective in oral tolerance induction compared to systemic administration of rat IL-10 [43]. 

In ConA-induced liver injury in mice, IL-10 seems to affect tolerance induction so that IL-10-knock out gene mice do not represent tolerance in ConA-induced liver injury [25]. KC and CD4+ CD25+ Treg can produce IL-10 and induce tolerance. A class of regulatory T cell subsets, Tr1, can secrete IL-10 to present suppressive effect in IL-10-dependent manner with or without TGF-β [44]. It seems that cellular source of IL-10 is not as important as its protective role in ConA-induced liver injury [45]. In lymphocytes, TGF-β signals can prevent autoimmune diseases. Transgenic mice whose liver expressed TGF-β were generated by Longenecker, *et al*. Although TGF-β level in the serum of transgene mice was the same as TGF-β in the wild mice, this TGF-β expression could not preserve TGF-β (–/–) from multi-organ failure in mice. In conclusion, TGF-β produced in autocrine fashion has an unavoidable role in preventing autoimmune disease [46]. TGF-β also has a role in converting conventional CD4+ T cells into CD4+ CD25+ FOXP3+ Tregs in the liver [47].

Proposed Mechanisms for Liver Transplant Tolerance 

Despite transplant rejections, there are recipients with long-surviving liver transplants, not taking immunosuppressive drugs. Such a graft survival is associated with liver immunological tolerance. 

In human, hyperacute rejection of liver transplants occurs rarely. Although the liver may compel acute rejection, the damaged liver parenchyma heals soon and graft loss occurs rarely after immunosuppressive therapy. In contrast to high frequency of renal chronic rejection, chronic rejection of liver transplants occurs rarely [48].

Bile duct injury, presence of eosinophils, portal and hepatic vein endothelium, inflammation and infiltration of lymphocytes to portal tracts are the signs of acute rejection, which is the most frequent rejection in liver transplantation. The most important mechanism of acute rejection is CD4+ T cell, CD8+ T cells and macrophages as the most noticeable cells in the infiltrates, although NK and B cells contribute to it as well.

MHC is the main target of acute rejection. Speed and result of transplant rejections depend on MHC type of the donor and recipient. Thirty percent of the liver non-paranchymal cells, and biliary epithelial cells express MHC-I and not MHC-II. Hepatocytes constitutively express MHC-I, not MHC-II. However, MHC-I over-expression and class II expression are induced by inflammatory conditions [1].

Role of Soluble Donor MHC-I Molecules

Soluble MHC induces donor-specific tolerance and plays a key role in transplantations. Secretion of soluble MHC-I molecules from liver transplants increases the graft survival. Both injection of genetically modified hepatocytes or usage of MHC-I encoding adenovirus can increase soluble donor MHC-I secretion, which results in liver allograft survival. Soluble MHC can induce tolerance by direct interaction with TCR, which leads the alloreactive T cells to apoptotic process [49]. Soluble MHC molecules can process and present donor peptides to T cells by APCs such as immature DCs, which act as tolerogenic DCs and induce partial tolerance. Also, mechanism of tolerance induction might directly bind soluble MHC to alloreactive antibodies [50]. Infusion of soluble donor MHC molecules that are bound to monoclonal antibody is more efficient in survival of allograft than injection of only soluble donor MHC because of more uptake of alo-MHC-antibody by APCs [51]. Although injection of soluble MHC can prolong the survival of liver transplants, it does not contribute to long-term tolerance of transplants for patients. 

Impact of Donor HLA-C Genotype

HLA-C is not considered very important in the transplantation process. Two groups of HLA (HLA-C1) and (HLA-C2), are recognized by the types of their ligation to 2D-killer immunoglobulin-like receptors, present on NK cells. Inhibitory activating ligation of HLA-C to KIRs depends on cytoplasmic tail length (s, activating) and (L, inhibitory). Although no obvious link was seen between HLA-C type or KIR genes with kidney transplants, acute rejection seems to be associated with genotype of KIR [52-54]. In 2008, a study showed the noticeable role of donor HLA-C genotype in liver transplant patients [55]. Patients whose donors were homozygous for an HLA-C2 allele had 26.5% greater survival of graft for 10 years compared with those with homozygote C1 alleles (p=0.001); this was 15.6% for heterozygous HLA-C2 allele compared with HLA-C1 patients (p=0.004). However, hepatic artery thrombosis has not been associated with donor HLA-C2 genotype; the incidence of late cirrhosis, and chronic rejection, was decreased in recipients of C2 donor livers. Inhibition of host NK cells by much more effective inhibitory signals of HLA-C2 (rather than HLA-C1) to NK cells results in decreased graft injury and inflammations [55]. 

Migratory Donor Cells

In migratory donor cells, as human blood transfusion experiments show, allograft survival is prolonged as the result of multiple blood transfusions before transplantation surgery. Comparing all immunosuppressive drugs, perioperative injection of blood containing donor cells is more efficient for tolerance induction as a consequence of transferring passenger donor cells to patients receiving grafts [56]. Organ transplant consists of parenchymal and non-parenchymal cells. Non-parenchymal hematopoietic stem cells and passenger leukocytes are capable of leaving transplanted graft to different parts of the recipient. Passenger leukocytes inducing DCs, B and T cells emigrate through the blood to secondary lymphoid tissues of the recipient [57, 58]. While hematopoietic stem cells migrate to recipients’ bone marrow, they lead to an increase in donor-derived cells such as DCs and T cells [59]. Passenger migratory donor cells may activate host T cells within lymphoid tissues defectively. This may kill T cells by neglecting them or result in long-term donor cell microchimerism, which acts as host T cells antagonists. Emigration of passenger cells from the graft results in liver transplant tolerance. The number of migratory donor cells depends on the size of transplanted organ. More migratory donor cells are available in larger grafts. Totally, by promoting the long-term microchimerism and increasing passenger donor cell number, tolerance would be enhanced [60].

Long-term Donor Cell Microchimerism 

Donor type of the microchimerism is defined as the presence of low population of donor-derived hematopoietic cells after solid organ transplantation. Microchimerism has been suggested as donor-specific graft tolerance induction. According to Starzl, *et al*, microchimerism is responsible for liver acceptance after transplantations. So donor cell microchimerism occurs in less than 1% in long-term surviving patients who receive liver transplants [61]. Although host *vs*. graft rejection (HVG) may abrogate the whole organ, GVH may occur as a consequence of severe immune suppression. Immune cellular interactions cause immunosuppression both in HVG and GVH. The graft can be accepted if these two directions’ responses balance. After months, GVH and HVG may stabilize and thus immunosuppressive regimens can be diminished [62]. It has not been clarified whether donor cell chimerism is the consequence of tolerance or cause of it. Long-term microchimerism occurs frequently in human transplantations but it is not related to graft integrity. Cell microchimerism can be observed both in patients receiving liver with an intrinsic tolerogenic capacity and the ones receiving other solid organ transplant [63]. Long-term microchimerism may not be crucial for transplant acceptance, but donor leukocytes play an essential role in induction stage of allograft tolerance [64]. It has been suggested that microchimerism is important to maintain tolerance, but it is still unclear if microchimerism induces tolerance by providing a constant reservoir of donor antigen result for T cell exhaustion or by directly killing alloreactive T cells [64]. 

## SUMMARY

Hepatic tolerance is accomplished both systemically and locally in the liver. 

Mice liver allograft acceptation needs no immunosuppression and will be occurred spontaneously.

Non-parenchymal and microenvironment cells have a main role in preventing the rejection of parenchyma cell.

It seems that hepatic tolerance act through a mixture of various non-redundant mechanisms, such as immune deviation, production and activation of regulatory T cells and tolerogenic molecules, like PD-L1, IL-10 and TGF-β. The controlling factors, which adjust the transition from hepatic tolerance to hepatic inflammation and back, are unknown, although it became obvious that innate immunity is a major regulator of this transition. In summary, liver transplants have multiple properties that make it different from other solid organ transplants, and these might justify the spontaneous acceptance. One of the outstanding capacities of liver is its spectacular regenerative ability and secretion of high levels of soluble MHC-I molecules, which prevent the liver damage caused by pre-existing alloantibodies soon after transplantation. At this time, the liver transplantation might participate in the activation of both donor-specific naive and effector T cells. Different mechanisms might happen simultaneously; certain events situate in the SLT, and others occur within the liver graft itself. Although the transplanted liver apparently induces peripheral deletion of naive and effector T cells, yet no evidence is found to propose that memory T cells are influenced. So, finding remedies that favor primary activation of allogeneic T cells in the liver and simultaneously inactivate pre-existing memory and effector T cells, which cross-react against the graft, will be an important concern for future treatments in transplantation of the liver and other solid organs. 

## References

[1] Tiegs G, Lohse WA (2010). Immune tolerance: What is unique about the liver. J Autoimmun.

[2] Racanelli V, Rehermann B (2006). The liver as an immunological organ. Hepatology.

[3] Carambia A, Herkel J (2010). CD4 T cells in hepatic immune tolerance. J Autoimmun.

[4] Sumpter TL, Abe M, Tokita D, Thomson AW (2007). Dendritic cells, the liver, and transplantation. Hepatology.

[5] Sass G, Shembade ND, Haimerl F (2007). TNF-induced inhibition of mitochondrial apoptosis is mediated by a 20-dependent down-modulation of Bax. J Immunol.

[6] Knolle P, Schlaak J, Uhrig A (1995). Human Kupffer cells secretes IL-10 in response to lipopolysaccharide (LPS) challenge. J Hepatol.

[7] You Q, Cheng L, Kedl RM, Ju C (2008). Mechanism of T cell tolerance induction by murine hepatic Kupffer cells. Hepatology.

[8] Roland CR, Mangino MJ, Duffy BF, Flye MW (1993). Lymphocyte suppression by Kupffer cells prevents portal venous tolerance induction: a study of macrophage function after intravenous gadolinium. Transplantation.

[9] Chen Y, Liu Z, Liang S (2008). Role of Kuppfer cells in the induction of tolerance of orthotopic liver transplantation in rats. Liver Transpl.

[10] Diehl L, Schurich A, Grochtmann R (2008). Tolerogenic maturation of liver sinusoidal endothelial cells promotes B7- homolog 1-dependent CD8‏ T cell tolerance. Hepatology.

[11] Knolle PA, Germann T, Treichel U (1999). Endotoxin down-regulates T cell activation by antigen-presenting liver sinusoidal endothelial cells. J Immunol.

[12] Limmer A, Ohl J, Kurts C (2000). Efficient presentation of exogenous antigen by liver endothelial cells to CD8+ T cells results in antigen-specific T cell tolerance. Nat Med.

[13] Winau F, Hegasy G, Weiskirchen R (2007). Ito cells are liver-resident antigen-presenting cells for activating T cell responses. Immunity.

[14] Yu MC, Chen CH, Liang X (2004). Inhibition of T cell responses by hepatic stellate cells via B7-H1-mediated T-cell apoptosis in mice. Hepatology.

[15] Strober W (2008). Vitamin A rewrites the ABCs of oral tolerance. Mucosal Immunol.

[16] Bamboat ZM, Stableford JA, Plitas G (2009). Human liver dendritic cells promote T cell hypo responsiveness. J Immunol.

[17] Benten D, Kumaran V, Joseph B (2005). Hepatocyte transplantation activates hepatic stellate cells with beneficial modulation of cell engraftment in the rat. Hepatology.

[18] Warren A, Le Couteur DG, Fraser R (2006). T lymphocytes interact with hepatocytes through fenestrations in murine liver sinusoidal endothelial cells. Hepatology.

[19] Herkel J, Jagemann B, Wiegard C (2003). MHC class II-expressing hepatocytes function as antigen-presenting cells and activate specific CD4+T lymphocytes. Hepatology.

[20] Sharpe AH, Wherry EJ, Ahmed R, Freeman GJ (2007). The function of programmed cell death 1 and its ligands in regulating autoimmunity and infection. Nat Immunol.

[21] Tang Q, Bluestone JA (2008). The FoxP3+regulatory T cell: a jack of all trades, master of regulation. Nat Immunol.

[22] Sakaguchi S, Yamaguchi T, Nomura T, Ono M (30). Regulatory T cells and immune tolerance. Cell.

[23] Longhi MS, Ma Y, Bogdanos DP (2004). Impairment of CD4(+)CD25(+) regulatory T-cells in autoimmune liver disease. J Hepatol.

[24] Lan RY, Cheng C, Lian ZX (2006). Liver-targeted and peripheral blood alterations of regulatory T cells in primary biliary cirrhosis. Hepatology.

[25] Erhardt A, Biburger M, Papadopoulos T, Tiegs G (2007). IL-10, regulatory T cells and Kupffer cells mediate tolerance in concanavalin A-induced liver injury in mice. Hepatology.

[26] Wei HX, Chuang YH, Li B (2008). CD4+ CD25+ Foxp3+ regulatory T cells protect against T cell-mediated fulminant hepatitis in a TGFbeta- dependent manner in mice. J Immunol.

[27] Abel M, Sène D, Pol S (2006). Intrahepatic virus-specific IL-10-producing CD8 T cells prevent liver damage during chronic hepatitis C virus infection. Hepatology.

[28] Bettelli E, Oukka M, Kuchroo VK (2007). Th-17 cells in the circle of immunity and autoimmunity. Nat Immunol.

[29] Lan RY, Salunga TL, Tsuneyama K (2009). Hepatic IL-17 responses in human and murine primary biliary cirrhosis. J Autoimmun.

[30] Nishimura T, Ohta A (1999). A critical role for antigen-specific Th1 cells in acute liver injury in mice. J Immunol.

[31] Jones DE, Palmer JM, Burt AD (2002). Bacterial motif DNA as an adjuvant for the breakdown of immune self-tolerance to pyruvate dehydrogenase complex. Hepatology.

[32] Klugewitz K, Blumenthal-Barby F, Schrage A (2002). Immunomodulatory effects of the liver: deletion of activated CD4+ effector cells and suppression of IFN-gamma-producing cells after intravenous protein immunization. J Immunol.

[33] Cabillic F, Rougier N, Basset C (2006). Hepatic environment elicits monocyte differentiation into a dendritic cell subset directing Th2 response. J Hepatol.

[34] Wiegard C, Wolint P, Frenzel C (2007). Defective T helper response of hepatocyte-stimulated CD4 T cells impairs antiviral CD8 response and viral clearance. Gastroenterology.

[35] Tsai SL, Liaw YF, Chen MH (1997). Detection of type 2-like T-helper cells in hepatitis C virus infection: implications for hepatitis C virus chronicity. Hepatology.

[36] Semmo N, Day CL, Ward SM (2005). Preferential loss of IL-2-secreting CD4+ T helper cells in chronic HCV infection. Hepatology.

[37] Fife BT, Bluestone JA (2008). Control of peripheral T-cell tolerance and autoimmunity via the CTLA-4 and PD-1 pathways. Immunol Rev.

[38] Rehermann B, Nascimbeni M (2005). Immunology of hepatitis B virus and hepatitis C virus infection. Nat Rev Immunol.

[39] Nakamoto N, Kaplan DE, Coleclough J (2008). Functional restoration of HCV-specific CD8 T cells by PD-1 blockade is defined by PD-1 expression and compartmentalization. Gastroenterology.

[40] Dong H, Zhu G, Tamada K (2004). B7-H1 determines accumulation and deletion of intrahepatic CD8(+) T lymphocytes. Immunity.

[41] Chen CH, Kuo LM, Chang Y (2006). In vivo immune modulatory activity of hepatic stellate cells in mice. Hepatology.

[42] Nakamoto N, Cho H, Shaked A (2009). Synergistic reversal of intrahepatic HCV-specific CD8 T cell exhaustion by combined PD-1/CTLA-4 blockade. PLoS Patho.

[43] Safadi R, Alvarez CE, Ohta M (2005). Enhanced oral tolerance in transgenic mice with hepatocyte secretion of IL-10. J Immunol.

[44] Jonuleit H, Schmitt E (2003). The regulatory T cell family: distinct subsets and their interrelations. J Immunol.

[45] Nelson DR, Lauwers GY, Lau JY, Davis GL (2000). Interleukin 10 treatment reduces fibrosis in patients with chronic hepatitis C: a pilot trial of interferon nonresponders. Gastroenterology.

[46] Longenecker G, Thyagarajan T, Nagineni CN (2002). Endocrine expression of the active form of TGF-beta1 in the TGF-beta1 null mice fails to ameliorate lethal phenotype. Cytokine.

[47] Luth S, Huber S, Schramm C (2008). Ectopic expression of neural autoantigen in mouse liver suppresses experimental autoimmune neuroinflammation by inducing antigen-specific Tregs. J Clin Invest.

[48] Stuart J, Knechtle MD, Kwun J (2009). Unique Aspects of Rejection and Tolerance in Liver Transplantation. Seminars in liver disease.

[49] Benseler V, McCaughan GW, Schlitt J (2007). The Liver: A Special Case in Transplantation Tolerance. The liver and transplantation tolerance.

[50] Hawiger D, Inaba K, Dorsett Y (2001). Dendritic cells induceperipheral T cell unresponsiveness under steady state conditions in vivo. J Exp Med.

[51] Sumimoto R, Kamada N (1990). Specific suppression of allograft rejection by soluble class I antigen and complexes with monoclonal antibody. Transplantation.

[52] Mandelboim O, Reyburn HT, Sheu EG (1997). The binding of NK receptors on HLA-C molecules. Immunity.

[53] Tran TH, Mytilineos J, Scherer S (2005). Analysis of KIR ligand incompatibility in human renal transplantation. Transplantation.

[54] Bishara A, Brautbar C, Zamir G (2005). Impact of HLA-C and Bw epitopes disparity on liver transplantation outcome. Hum Immunol.

[55] Hanvesakul R, Spencer N, Cook M (2008). Donor HLA-C genotype has a profound impact on the clinical outcome following liver transplantation. Am J Transplant.

[56] Siemionow M, Agaoglu G (2005). Role of blood transfusion I transplantation: a review. J Reconstr Microsurg.

[57] Bishop GA, Sun J, DeCruz DJ (1996). Tolerance to rat liver allografts: III. Donor cell migration and tolerance-associated cytokine production in peripheral lymphoid tissues. J Immunol.

[58] Schlitt HJ, Kanehiro H, Raddatz G (1993). Persistence of donor lymphocytes in liver allograft recipients. Transplantation.

[59] Medawar PB (1944). The behavior and fate of skin autografts and skin homografts in rabbits. J Anat.

[60] Strober W (2008). Vitamin A rewrites the ABCs of oral tolerance. Mucosal Immunol.

[61] Starzl TE, Demetris AJ, Trucco M (1992). Systemic chimerism in human female recipients of male livers. Lancet.

[62] Starzl TE, Lakkis FG (2006). The unfinished legacy of liver transplantation: emphasis on immunology. Hepatology.

[63] Starzl TE, Demetris AJ, Trucco M (1993). Chimerism and donor-specific nonreactivity 27 to 29 years after kidney allotransplantation. Transplantation.

[64] Hamano K, Rawsthorne MA, Bushell AR (1996). Evidence that the continued presence of the organ graft and not peripheral donor microchimerism is essential for maintenance of tolerance to alloantigen in vivo in anti-CD4 treated recipients. Transplantation.

